# Genetic Inertia in Urban Populations of the Common Toad (*Bufo bufo*): Evidence from Nuclear and Mitochondrial DNA

**DOI:** 10.3390/ani16131983

**Published:** 2026-06-27

**Authors:** Anna Sztencel-Jabłonka, Aleksandra G. Bilska, Barbara Bujalska, Joanna Mazgajska, Tomasz D. Mazgajski, Veronika Hrabovcová Sládkovičová, Zbigniew Borowski, Anna Tereba, Michal J. Dabrowski

**Affiliations:** 1 Museum and Institute of Zoology, Polish Academy of Sciences, Twarda 51/55, 00-818 Warsaw, Poland; abilska@miiz.waw.pl (A.G.B.); jmazgajska@miiz.waw.pl (J.M.); tmazgajski@miiz.waw.pl (T.D.M.); 2 Institute of Computer Science, Polish Academy of Sciences, Jana Kazimierza 5, 01-248 Warsaw, Poland; 3 Doctoral School of Molecular Medicine, Medical University of Lodz, Pl. Hallera 1, 90-647 Lodz, Poland; 4 Department of Zoology, Faculty of Natural Sciences, Comenius University in Bratislava, Mlynská Dolina, Ilkovičova 6, 842 15 Bratislava, Slovakia; 5 Forest Research Institute, Sękocin Stary, Braci Leśnej nr 3, 05-090 Raszyn, Poland; z.borowski@ibles.waw.pl (Z.B.); a.tereba@ibles.waw.pl (A.T.)

**Keywords:** amphibians, *Bufo bufo*, genetic erosion, genetic landscape, MCFS-ID, microsatellites, mtDNA, urban fragmentation, Warsaw

## Abstract

Urban-driven landscape fragmentation is a major factor contributing to amphibian population decline yet its genetic consequences remain incompletely understood. We examined the genetic structure and variability of the common toad (*Bufo bufo*) in Warsaw, Poland, using seven nuclear microsatellite loci and mitochondrial cytochrome b sequences (800 bp). Samples (N = 97) were collected from six breeding sites representing contrasting urban habitats on both banks of the Vistula River. Genetic analyses revealed low but significant population differentiation, elevated inbreeding coefficients, and a heterozygosity deficit despite relatively high effective population sizes. This pattern is consistent with post-fragmentation genetic inertia, in which demographic buffering temporarily maintains genetic diversity despite reduced connectivity. Consequently, contemporary genetic patterns may underestimate the extent of ecological isolation imposed by urban infrastructure. Mitochondrial analyses identified seven haplotypes, including six previously unreported, forming a star-like network topology indicative of a single postglacial colonization event. The Vistula River did not constitute a significant barrier to gene flow, based on pairwise F_ST_ and hierarchical AMOVA. Supervised feature selection (MCFS-ID) identified specific alleles and body mass as key discriminants among sites, suggesting emerging responses to local urban conditions. Our results indicate that urban common toad populations can retain substantial genetic variation despite fragmentation, although continued isolation may ultimately increase inbreeding and genetic drift.

## Simple Summary

Urbanization fragments natural habitats and may isolate wildlife populations. Amphibians are particularly vulnerable because they depend on both aquatic and terrestrial environments and often have limited dispersal abilities. We investigated how urbanization affects the genetic diversity of the common toad (*Bufo bufo*) in Warsaw, Poland. Using nuclear and mitochondrial genetic markers, we analyzed individuals from six breeding sites located across different parts of the city, including both sides of the Vistula River. Despite evidence of reduced connectivity and some genetic differentiation between sites, the populations still retained relatively high genetic diversity. This suggests that the genetic effects of habitat fragmentation may emerge more slowly than ecological isolation itself. We also found that the Vistula River does not represent a major barrier to gene flow for common toads in Warsaw. Several genetic variants and body mass differences were associated with particular sites, indicating possible local responses to urban environmental conditions. Our results show that urban amphibian populations may temporarily preserve genetic diversity despite increasing habitat fragmentation. However, continued isolation could eventually lead to greater inbreeding and loss of adaptive potential. Maintaining ecological connectivity, for example, through green corridors and protection of breeding habitats, may therefore be important for the long-term conservation of amphibians in cities.

## 1. Introduction

Urban expansion is a dominant feature of contemporary landscapes, leading to progressive fragmentation of natural habitats and the reduction of green areas to isolated remnants within urban matrices [1,2]. As a consequence, populations of many species become increasingly spatially separated, often functioning as metapopulations characterized by local colonization and extinction dynamics [3,4]. In organisms with limited dispersal ability, however, restricted movement between habitat patches may shift this balance toward local extinctions rather than recolonization [5]. Such isolation can have cascading effects across biological levels, influencing individual condition and fitness, altering population dynamics, and ultimately driving genetic differentiation among populations [6].

Urban fragmentation affects the geometry and quality of habitat patches (size, structure), influencing key population traits such as dispersal, density, and life history [7,8,9]. Isolation resulting from urban fragmentation restricts gene flow between habitat patches, increasing the risk of genetic diversity loss and local extinction. Moreover, rapid fragmentation can lead to population divergence [7], induce genetic structure shifts with minimal delay [10,11,12], and ultimately reduce genetic variation over a longer period [13].

Existing studies on urban vertebrate populations are strongly biased toward birds and mammals [14,15]. In contrast, amphibians—recognized as the most threatened vertebrate class and particularly sensitive to landscape resistance and matrix composition [16], remain markedly underrepresented in urban fauna research [14]. Their dual aquatic-terrestrial lifestyle makes them highly sensitive to anthropogenic barriers, particularly roads, which inflict high traffic mortality and limit dispersal [17,18,19,20,21]. Understanding how the urban landscape matrix mediates gene flow is essential, as fine-scale habitat requirements and susceptibility to pollution and degradation are directly linked to the surrounding landscape structure [5,8,22,23]. Studies have reported a negative relationship between urbanization levels and amphibian population characteristics [16,24,25,26,27].

Habitat fragmentation does not necessarily result in immediate genetic differentiation. Genetic responses to landscape change may lag behind ecological isolation, creating a temporal mismatch between contemporary habitat connectivity and population genetic patterns [7,28]. During this period, populations may retain relatively high genetic diversity and only weak genetic structure despite reduced connectivity, a phenomenon often described as genetic inertia or time-lag effects.

The common toad (*Bufo bufo*), which is abundant in most European cities, is a suitable model for studying amphibian ecological functioning in urban ecosystems [29,30]. It can breed in small garden ponds and larger park water bodies, exhibits strong breeding site fidelity [31,32], and can migrate up to 3 km [33,34]. Spring migration in urban environments is frequently characterized by mass mortality on roads [20,35,36,37,38], which, together with the naturally moderate vagility of the species, contributes to population differentiation in fragmented habitats [39].

Under persistent urban pressures, common toad populations continue to decline. This trend, first documented in the 1990s [40], has been associated with habitat degradation, pollution, and altered microclimates, contributing to increased developmental abnormalities, potentially linked to reduced genetic heterozygosity [30]. Urban conditions also shape amphibian physiology, phenology, and reproductive success [29,41,42,43]. Despite some degree of adaptive plasticity, the long-term consequences of these processes for population dynamics and genetic structure in urban toad populations remain insufficiently understood. Due to limited dispersal ability and the increasing number of barriers separating breeding sites, local populations of the common toad become progressively isolated and may eventually undergo local decline or extinction. In Poland, studies conducted across multiple major cities have consistently documented reduced connectivity and deteriorating population parameters under urban fragmentation [44,45,46,47]. In Warsaw, 39% of known breeding sites have been lost over the past two decades, and the remaining sites are generally located at considerable distances from the city center (on average over 8 km; [42]). Such spatial isolation may limit individual exchange among sites, potentially influencing patterns of gene flow and shaping the genetic structure of urban populations. These characteristics make Warsaw a useful system for examining whether contemporary ecological isolation is already reflected in population genetic structure.

In previous research on *B. bufo* populations in cities, a trend of population size decline and reduction in genetic variation was demonstrated [48,49]. Lower genetic richness of urban populations than that of rural populations has been found in Europe [30], where city development has been indicated as a barrier to the migration of individuals. Lower gene flow and deteriorating population dynamics have also been reported [50,51]. Also in the European green toad (*Bufotes viridis*) populations, gene flow was significantly restricted within urbanized landscapes—characterized by dense development and the loss of natural wetland corridors adjacent to rivers—compared to nearby rural populations, despite shorter geographic distances between urban sites [39]. On the other hand, recent study [52] found limited evidence for strong genetic differentiation or local adaptation across rural–urban gradients in *B. bufo*, suggesting that urbanization does not always lead to detectable erosion of genetic diversity, at least over shorter temporal scales or where habitat connectivity persists (likely because of persisting natural habitats within or around cities). Together, these findings indicate that the genetic consequences of urbanization may vary among landscapes and may not always emerge immediately following habitat fragmentation.

The role of large unregulated rivers in shaping urban amphibian population structure remains poorly understood. While even small streams can act as dispersal barriers [53,54], the role of major hydrologic boundaries is less frequently analyzed. Evidence from other taxa suggests that such features can profoundly shape urban genetic landscape. For example, gene flow in European green toad (*Bufotes viridis*) populations was significantly restricted within urbanized landscapes compared to nearby rural populations, despite shorter geographic distances between urban sites. This pattern was associated with dense urban development and the loss of wetland corridors adjacent to rivers [39]. Notably, in *Bufotes viridis* populations from Cologne, while microsatellite analyses revealed no clear genetic differentiation across the Rhine River, mitochondrial DNA data identified a haplotype exclusive to the eastern bank, suggesting that the Rhine acts as a partial or historical barrier to gene flow in this species [55]. In this context, it remains unclear whether the Vistula functions as a primary phylogeographic divide for *B. bufo* or merely a selective filter with lower resistance than the surrounding anthropogenic matrix of roads and dense infrastructure.

This study examined the *B. bufo* population in Warsaw, Poland’s capital city, characterized by the Vistula River’s division into distinct left and right banks. Warsaw therefore represents a particularly informative urban system in which a major river barrier occurs simultaneously with dense urban infrastructure. This configuration allows the relative contribution of riverine and urban barriers to be evaluated within the same metropolitan landscape. The right bank, a Natura 2000 site, features natural riparian ecosystems and serves as an essential ecological corridor [56]. In contrast, the left bank has been extensively modified for urban expansion [57].

Building on previous amphibian surveys in Warsaw [47,58], we selected *Bufo bufo* breeding ponds, including sites and clusters of ponds separated by substantial distances, to conduct a comprehensive genetic assessment. Given the relatively recent history of urban expansion in Warsaw and the limited knowledge regarding the role of large riverine barriers in urban amphibian populations, we did not formulate strong directional predictions. Instead, we used complementary nuclear and mitochondrial markers to evaluate patterns of genetic diversity, differentiation, and population structuring across the city. Using a multilocus approach, we aimed to: (1) assess overall genetic diversity and inbreeding levels across urban populations; (2) evaluate the extent of genetic differentiation among spatially isolated breeding sites; and (3) test for signatures of population structuring potentially associated with urban fragmentation, including the potential role of the Vistula River as a barrier to gene flow.

## 2. Materials and Methods

### 2.1. Study Area

The study was conducted in Warsaw, a major urban center characterized by a highly fragmented and heterogeneous habitat matrix. The city is traversed by the unregulated Vistula River and an extensive network of roads and urban infrastructure, which together form a high-resistance matrix for amphibian dispersal. Herpetological research in the region has a well-established history, with detailed studies carried out as early as the 1990s [58,59,60,61]. During surveys conducted between 1992 and 1994, the common toad (*Bufo bufo*) was recorded in 30.3% of the monitored ponds (N = 76) [59]. However recent surveys of breeding sites of the common toad (*Bufo bufo*) in Warsaw have documented a substantial decline in the number of occupied sites, with only 16 localities currently confirmed, often supporting small numbers of individuals [42]. These remaining sites are predominantly located at the periphery of the city and form distinct spatial groupings that can be broadly categorized into six regions: central, northern, north-eastern, western, southern, and eastern (beyond the Vistula River) (cf. Figure 1 in [42]).

Based on this distribution, six breeding sites were selected for the present study. Given their spatial arrangement, these sites can be considered to represent four main groups: a northern group (NW1: 52.245 N, 20.893 E; NW2: 52.243 N, 20.900 E), including the nearby western site (W: 52.218 N, 20.911 E); a southern group (SE1: 52.141 N, 21.075 E; SE2: 52.133 N, 21.042 E); and an eastern site located on the opposite bank of the Vistula River (E: 52.205 N, 21.124 E).

**Figure 1:**
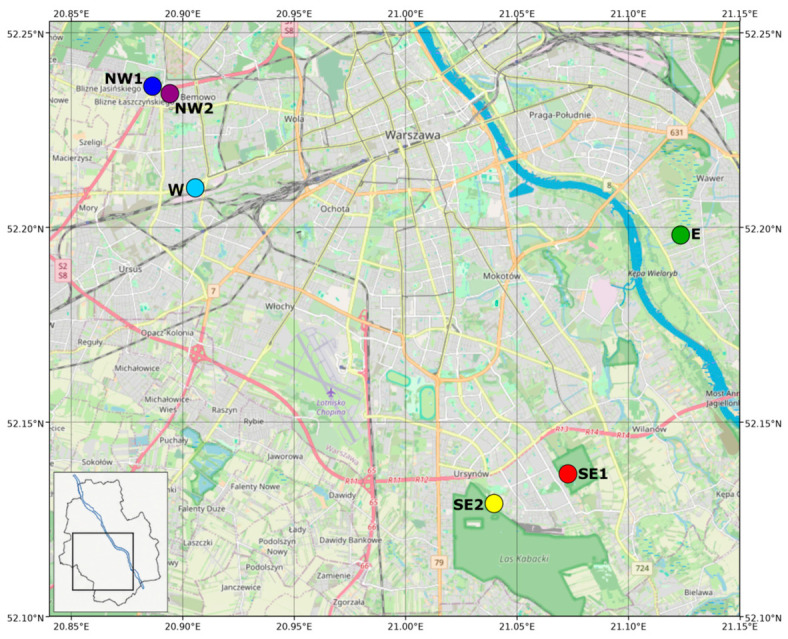
Common toad sampling sites in Warsaw. Colored circles indicate sampling localities, with each color corresponding to a different site. The map shows the sites in relation to the Vistula River, major green areas, urban barriers, and transportation corridors. A detailed interactive map is provided in the Supplementary Material (File S1). Author-generated map created using Leaflet.js v1.9.4. and Python v3.14.6 (code development assisted by ChatGPT, OpenAI GPT-5.5).

These sites were selected because population sizes were sufficiently large to allow sampling, whereas sites located in the city center and parts of the northern region supported only isolated individuals and were therefore excluded from genetic analyses. Jezioro Żabie (E), located on the right bank of the Vistula River, was specifically included to evaluate the potential barrier effect of the river (Figure 1). The spatial configuration of the selected sites provides a suitable framework to test whether habitat fragmentation and resulting isolation influence genetic structure and diversity. In particular, the inclusion of two geographically proximate northern breeding sites (NW1 and NW2), separated primarily by urban infrastructure, enabled assessment of local-scale differentiation, whereas comparisons among more distant sites distributed across Warsaw allowed evaluation of broader regional patterns of genetic structure. All sites consisted of typical urban ponds embedded within built-up areas. An interactive map of the sampling sites, created using Leaflet.js v1.9.4., is provided in the HTML format as Supplementary Material (File S1).

### 2.2. Field Work

The water bodies were monitored for breeding activity in spring. Individuals were captured, weighed, and measured under laboratory conditions. Each toad was PIT-tagged, and two phalanges of the fourth digit on the right hind limb were collected from the selected individuals for skeletochronological analysis. All amphibians were subsequently released at the capture sites. Common toads were sampled during the breeding seasons of 2011 (W: *n* = 11; E: *n* = 19) and 2012 (W: *n* = 7; E: *n* = 2; SE1: *n* = 20; SE2: *n* = 19; NW1: *n* = 9; and NW2: *n* = 10). Samples were collected as part of a study that investigated the effect of urbanization on toads’ condition [42,62]. The genetic analyses presented here were conducted using tissue material obtained during that study. Consequently, the number of sampled individuals and their distribution among breeding sites reflected the design and field constraints of the original project rather than a sampling scheme developed specifically for the present genetic investigation. Sampling efforts aimed to achieve a balanced sex ratio, despite field observations indicating a male bias (details in [62]). The final dataset included 58 males (59.79%) and 39 females (40.21%).

### 2.3. Genetic Analyses

#### 2.3.1. Nuclear DNA

Toe skin tissue stored in 70% ethanol was used for molecular analyses. The tissue originated from phalange samples previously collected for skeletochronological analyses [42,62]. Sampling and animal handling procedures were approved by the Local Ethical Committee for Animal Experiments in Warsaw (decision no. 1040/2009) and the General Directorate for Environmental Protection (decision no. DOOŚ-OA.4200/II-50/2398/10/JRO). Consequently, no additional sampling of animals was required, as the present study utilized biological material already collected for previous research purposes. DNA was extracted from 97 individuals using the NucleoSpin Tissue kit (Macherey Nagel, Düren, Germany), QIAamp DNA Mini Kit (Qiagen, Hilden, Germany), and Genomic Mini kit (A&A Biotechnology, Gdańsk, Poland), following the manufacturer’s protocols. Seven highly polymorphic microsatellite loci: Bbuf11, Bbuf14, Bbuf24, Bbuf47, Bbuf49, Bbuf62, Bbuf63 [63] were amplified using PCR following the authors’ protocol on a Veriti^®^ Thermal Cycler (Applied Biosystems, Foster City, CA, USA). Isolated DNA was stored at −20 °C for short-term and −80 °C for long-term storage. The amplification products were analyzed using a CEQ 8000 sequencer (Beckman Coulter, Fullerton, CA, USA).

#### 2.3.2. mtDNA

An 800 bp fragment of the mitochondrial cytochrome b gene (mtDNA) was amplified using the primers Cyt Bufo F and Cyt Bufo R [64] under standard amplification conditions modified from [51]. Excess unincorporated primers were removed using a Clean-Up Purification Kit (A&A Biotechnology, Gdansk, Poland). Sequencing reactions were carried out in accordance with the standard protocol of BigDye Terminator v3.1 Ready Reaction Mix (ThermoFisher Scientific, Waltham, MA, USA). The cytochrome *b* mtDNA regions were sequenced using both primers on an ABI 3500 Genetic Analyzer (Applied Biosystems, Foster City, CA, USA).

### 2.4. Analysis of Population Genetic Structure Based on Nuclear DNA

To assess whether breeding sites differ genetically from one another, a set of complementary clustering analyses was performed. Genetic clusters were identified using Discriminant Analysis of Principal Components (DAPC) [65] implemented in the “adegenet” package version 2.1.10 [66] for R software v. 4.5.1 [67]). To choose the optimal number of principal components (PCs) in the DAPC analysis, we tested 30 different cases using the optim.a.score function. To avoid overfitting, 14 PCs were chosen, which also covered over 90% of the cumulative variance, and the find.clusters function was used to identify the number of clusters and selected based on the Bayesian Information Criterion (BIC). To describe clusters, we ran DAPC with the original sampling site labels and with individuals divided into clusters using 14 PCs in both cases.

Genetic clustering was additionally assessed using BAPS [68] with 10,000 iterations, snapclust function (“adegenet” package) based on Expectation-Maximization (EM) algorithm with default parameters and STRUCTURE 2.3.4 [69,70,71]. In the analysis, we set an admixture model with correlated allele frequencies, incorporating the sampling location as prior information. Each run consisted of 100,000 Markov chain Monte Carlo (MCMC) iterations, with 50,000 discarded as burn-in, and 10 replicates were performed for each value of K (ranging from 1 to 8). To identify the optimal number of clusters, the Evanno method [72] was used via STRUCTURE Harvester [73]. Results were visualized using the compoplot function and “ggplot2” package version 4.0.0 [74] in R. To calculate genetic variability, the “diveRsity” version 1.9.90 [75] package in RStudio v.2025.5.1.513 was used. For individuals from each sampling site and designated genetic cluster, we computed several genetic parameters using the divBasic function: number of alleles (Na), observed (Ho) and expected (He) heterozygosity, and number of private alleles (No. Private Alleles), allelic richness (Ar), deviation from Hardy–Weinberg equilibrium (HWE), coefficient of inbreeding (F_IS_). The genetic distance (F_ST_) values were computed to assess genetic differentiation as a proxy for restricted gene flow mediated by the urban landscape. F_ST_ was computed between localities and clusters using the basic.stats function from the “hierfstat” package version 0.5.11 [76]. Pairwise F_ST_ significance was assessed using one-sided permutation tests with 9999 permutations per comparison. Because 15 pairwise comparisons were performed, *p*-values were corrected using the Bonferroni method. Results were considered significant at Bonferroni-adjusted *p* < 0.05, equivalent to raw *p* < 0.00333. FDR-BH-adjusted *p*-values were also reported for comparison. Isolation by distance was tested using a Mantel test between the pairwise F_ST_ matrix and the matrix of planar Euclidean geographic distances, with Pearson correlation and 9999 permutations. The Vistula River barrier hypothesis was tested using hierarchical AMOVA, with populations grouped by riverbank using a Bank/Population hierarchy. Confidence intervals for allelic richness, F_IS_, observed heterozygosity, and expected heterozygosity were estimated using a leave-one-locus-out jackknife procedure across seven microsatellite loci. We used NeEstimator V2.1 [77] and LDNe software v1.31 [78] to compute the effective population size (Ne) using the linkage disequilibrium method. Effective population size was estimated using two commonly applied allele-frequency thresholds (Pcrit = 0.02 and Pcrit = 0.05), which reduce the influence of rare alleles on linkage disequilibrium-based Ne estimation while facilitating comparison with previous studies [77,78]. Ne estimates were used to assess the demographic health of populations within these fragmented landscape patches. To look for signals of recent bottlenecks, we used the one-tailed Wilcoxon test [79] implemented in Bottleneck 1.2.02 software [80] using a Stepwise Mutation Model (SMM) and a Two-Phase Model (TPM) with 90% SMM.

To compare the differences in variables for the designated genetic clusters, we used parametric (ANOVA) or nonparametric (Kruskal–Wallis) statistical tests, depending on data normality, using the Shapiro–Wilk test. The chi-squared test was used for the analysis of categorical data. The obtained 97 sequences (mtDNA, an 800 bp fragment of cytochrome b sequence) were aligned using the Clustal W algorithm implemented in BioEdit [81]. A network of haplotypes was constructed using the “pegas” package version 1.3 in R [82]. Haplotype diversity (Hd), average number of differences (K), nucleotide diversity (Pi), and pairwise genetic distances between localities (F_ST_) were calculated using DnaSPv5 [83].

### 2.5. Feature Selection Analysis Integrating Genetic, Ecological and Morphological Traits

Based on the available ecological data collected covering individuals from this study (See details in: [62]), we included them in our genetic analyses and created a database with additional parameters, such as body weight, total body length, tibia length, or information related to mating behavior and reproduction occurrence of amplexus or spawning in females (Table S1). Our goal was to check whether there were features that significantly distinguished the examined sampling sites/clusters by creating a ranking of toad features (ecological, morphological, and genetic data), and to determine which of the studied traits are informative and non-informative in this respect, revealing the most variable classification attributes and possible interdependencies among the informative features. Morphological and ecological variables were included as complementary descriptors of population differentiation, allowing us to assess whether patterns of site discrimination identified from genetic data were accompanied by phenotypic differences among individuals inhabiting different urban environments. For this purpose, the Monte Carlo Feature Selection and Interdependency Discovery (MCFS-ID) algorithm [84] implemented in the R package “rmcfs”version 1.3.6 [85] was used. The cutoff point for significant versus non-significant features was assessed using the permutation method implemented in the rmcfs package. Following the package default settings, 20 permutations were used to determine the significance threshold. Analysis was performed with grouping (i) into sampling sites, (ii) into designated genetic clusters of individuals, and (iii) by gender using the rmcfs function and visualized on plots.

## 3. Results

### 3.1. Genetic Variability Based on Nuclear DNA

To characterize overall population genetic parameters and to provide a basis for comparing variation among breeding sites, we estimated basic overall population genetic parameters. The mean number of alleles ranged from 4 to 7, and allelic richness ranged from 3.72 to 5.23 (Table 1). Heterozygosity deficit and moderate to high positive F_IS_ values were detected at every sampling site, except for the SE2 locality, where F_IS_ equaled 0.005. This locality was also the only one with a higher observed than expected heterozygosity. The lowest genetic variability was detected in the northernmost locality, NW2, which also exhibited the highest inbreeding coefficient. To assess whether recent demographic declines associated with habitat fragmentation have left detectable genetic signatures, we tested for bottlenecks. However, we did not detect any signs of bottlenecks at the sampling sites using either the TPM or SMMs. Moreover, the estimated effective population size (Ne) was mostly large (with “infinite” estimates indicating no upper bound) or relatively high, with the exception of NW2, where Ne = 36.50 using both model assumptions (Table 1). Negative or infinite Ne estimates were interpreted as indicating insufficient information to define an upper bound for effective population size rather than biologically negative population sizes. Following [77,78], the lower confidence limits were therefore considered the most informative estimates in such cases.

**Figure 2:**
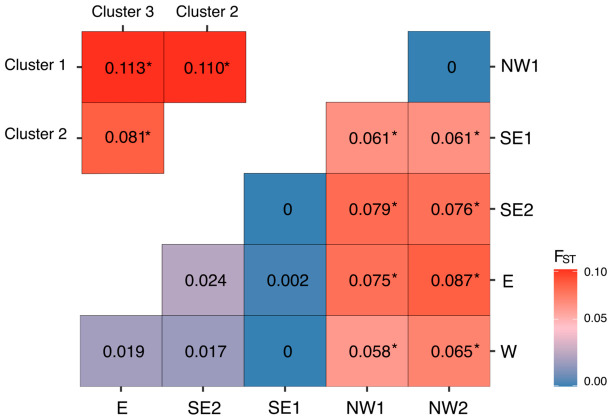
Pairwise genetic differentiation (F_ST_) among sampling sites and genetic clusters. Values with asterisk indicate comparisons significant after Bonferroni correction (*p* < 0.05) for 15 tests. Exact *p*-values are provided in Table S2.

Interestingly, significant pairwise genetic distances (F_ST_) were detected between locations, but with low values (Figure 2). Two northern sites (NW1 and NW2) had the highest values of pairwise F_ST_ with other sites, whereas the genetic distance between them was zero. This pattern indicates a shared genetic composition between the northern sites and their differentiation from the remaining breeding sites, a trend that was also suggested, albeit less clearly, by clustering analysis. After Bonferroni correction for 15 pairwise comparisons, 8 comparisons remained statistically significant. Exact raw *p*-values, Bonferroni-adjusted *p*-values, and FDR-BH-adjusted *p*-values are provided in Table S2. The Mantel test showed a positive but non-significant relationship between genetic and geographic distances (Mantel r = 0.4699, *p* = 0.0653), indicating only a weak, non-significant tendency toward isolation by distance. Hierarchical AMOVA did not reveal a significant Vistula-bank effect (Table S3). The between-bank component explained only 0.6935% of total genetic variation and was not significant (Phi-Bank-total = 0.0069, *p* = 0.3453). Taken together, these results reveal a consistent pattern across sampling sites: significant heterozygosity deficits and positive F_IS_ values, accompanied by low but significant pairwise genetic differentiation (F_ST_) and the absence of recent bottleneck signatures. This combination suggests that local effects of habitat fragmentation and restricted dispersal are already reflected in within-population genetic parameters, whereas stronger among-population divergence remains weakly developed. Such a pattern is consistent with the genetic expectations of a post-fragmentation genetic inertia (time-lag effect), explored further in the Discussion.

The overall genetic variability within the identified clusters was similar, and no significant differences were found (except for Ne values). The allelic richness ranged from 7.21 to 8.25, and the number of alleles ranged from 7.57 to 8.85, with the lowest values observed in cluster 2 (Table 2). No recent genetic bottlenecks were detected under either the SMM or TPMs. The estimates of Ne were relatively high, with the highest value for cluster 1. The expected heterozygosity in all three cases was higher than the observed heterozygosity. A heterozygosity deficit was found, resulting in positive F_IS_ values. Significant but relatively low pairwise genetic distances (from 0.08 to 0.11) were detected between all clusters (Figure 2).

### 3.2. Population Genetic Structure Reflected by Nuclear DNA

**Figure 3:**
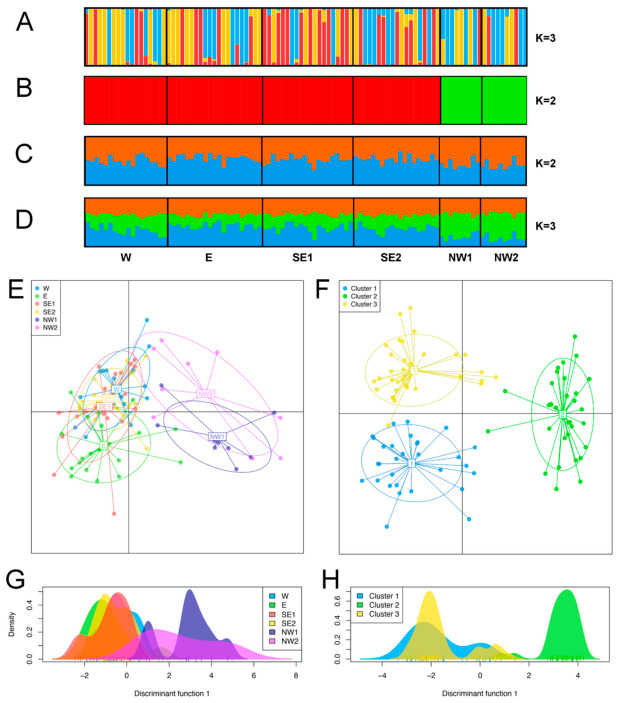
Population structure inferred using multiple clustering approaches: (**A**) snapclust revealed K = 3; (**B**) BAPS with K = 2; (**C**) STRUCTURE for K = 2; and (**D**) STRUCTURE for K = 3. Scatterplots from DAPC showing individuals from (**E**) the six sites with the variance explained by the first two discriminant functions (66.80% and 18.28%, respectively) and (**F**) the three clusters revealed by snapclust with the variance explained by the first two discriminant functions (77.62% and 22.36%, respectively). Density plots for discriminant function 1 for (**G**) sites and (**H**) clusters. The proportion of conserved variance was 0.725 for the clusters.

In line with our sampling design, we expected the emergence of three to four spatially coherent genetic clusters corresponding to the main site groupings. Genetic clustering analyses revealed varying population structures. The find.clusters function identified the optimal grouping of individuals into three clusters (K = 3; Figure S1). Using the snapclust function with K set to 3, individuals from all sampling sites showed substantial admixture among the inferred clusters (Figure 3A). Although the northern sites (NW1 and NW2) exhibited a somewhat different cluster membership profile compared with the remaining localities, individuals were not consistently assigned to clearly separated groups reflecting the geographic distribution of sites or their spatial proximity.

In contrast, the Bayesian Analysis of Population Structure (BAPS) indicated K = 2 as the most likely genetic structure (Figure 3B), clearly grouping the northernmost sites (NW1 and NW2) into one cluster and the remaining sites into the other. This result suggests a potential distinctiveness of the northern populations relative to the rest of the study area. Despite its geographic proximity to NW1 and NW2, site W clustered with the remaining sites, suggesting substantial genetic similarity among geographically separated localities, including sites located on opposite banks of the Vistula River (Figure 1).

However, the results obtained from STRUCTURE (Figure 3C) based on the ΔK approach also indicated K = 2 as the most likely genetic structure (ΔK = 3.4), which is the lowest number of clusters supported by the ΔK approach. Notably, this pattern did not consistently support the distinctiveness of the northern sites. Moreover, the mean individual assignment probabilities to the two inferred clusters were nearly equal (0.501 and 0.499, respectively), indicating limited differentiation. Conversely, the mean log-likelihood [LnP(K)] returned K = 1 as optimal (based on 50 out of 50 iterations, mean(lnProb) = −2034.4; mean (similar score) = 1.0), supporting a scenario of weak population structure. However, when examining the K = 3 scenario from STRUCTURE, the northern sites displayed some degree of differentiation (marked in green; Figure 3D). The DAPC also supported partial differentiation of the northern sites (Figure 3E). In addition, the easternmost population (E), separated by the Vistula River, showed a slight separation from the remaining sites (Figure 3G). The DAPC based on the three inferred clusters resulted in a good separation of the three proposed clusters (Figure 3F,H).

Overall, the results are not fully consistent across methods and indicate a weak and context-dependent population structure. While some analyses (BAPS, DAPC, and STRUCTURE at K = 3) suggest partial differentiation of the northern sites, this pattern is not robustly supported by all approaches and is not reflected in the most parsimonious clustering solutions (K = 1–2).

### 3.3. Features Differentiating Sampling Sites and Clusters

The MCFS-ID analysis integrated 86 input features, including genetic (alleles), ecological (sex, amplexus, female spawn), and morphological data (body weight, total body length, and tibia length; Table S1). The aim was to identify the most discriminant features between sites, clusters, and sex. Inter-population differentiation was best explained by five significant features (Figure S2A): one allele at the Bbuf49 locus, body weight, two alleles at the Bbuf63 locus, and tibial length. Notably, the top-ranked feature, allele 186 at the Bbuf49 locus, also showed the highest loadings (coefficients of the alleles used in the linear combination) in the DAPC analysis (Figure S3). Additionally, cluster differentiation was characterized by seven significant genetic features (Figure S2B): three alleles at the Bbuf62 locus, two at the Bbuf63 locus, and two at the Bbuf47 locus. These features were distinct from those differentiating the sites. Sex differentiation was, as expected, primarily attributed to ecological and morphological features (body weight and spawning) rather than genetic variables (Figure S2C).

Based on the top features returned by the MCFS-ID rankings, individuals were classified into sites, clusters, and sex. The classification-weighted accuracy (wacc) varied depending on the grouping criteria. For sampling sites, k-Nearest Neighbors (kNN) showed the highest weighted accuracy (wacc = 0.443); while seemingly moderate, this significantly exceeds the random accuracy for six sites 0.167), suggesting substantial discriminant power of the identified significant features (Table S5). More importantly, the cluster and sex classifications reached even higher wacc (>0.9), confirming strong cluster separation and clear sexual dimorphism. The selected set of significant features appeared to be optimal for each group (sampling sites, clusters, and sex); hence, increasing their number did not influence the wacc results, irrespective of the algorithm applied (Figure S4).

### 3.4. mtDNA Diversity of Urban Individuals

We identified seven haplotypes within the cytochrome b fragment (800 bp) in the analysed group of 97 individuals. Haplotype H1 (PQ463790) exhibited 100% identity to the reference mitochondrial genome of *B. bufo* from Denmark (MN 122891.1) deposited in GenBank. The remaining six haplotypes (PQ463791–PQ463796) are reported here as novel sequences and showed high similarity to the reference sequence, with identity ranging from 99.75 to 99.88% (one to two substitutions; Table S6).

**Figure 4:**
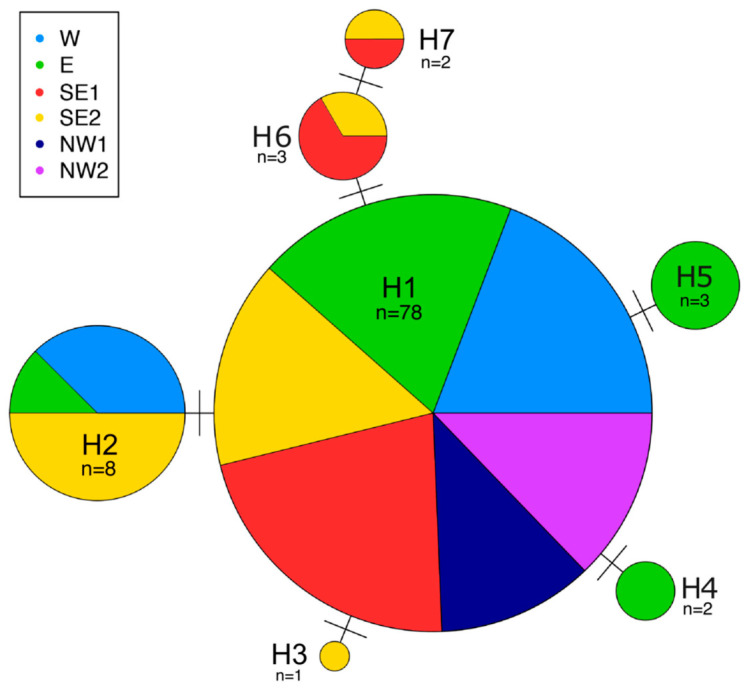
Network of seven haplotypes of common toads found in six localities in Warsaw.

Most individuals (80%) shared one dominant haplotype, which was found in all examined localities. Four sites were characterized by more than one haplotype (Table 3). SE2 was characterized by the highest number of haplotypes (n = 5), as well as the highest haplotype diversity (Hd = 0.579), average number of differences (K = 0.760), and nucleotide diversity (Pi = 0.00095). The haplotype network (Figure 4) exhibited a star-like topology. Nucleotide diversity was low at all sites (Table 3), as five haplotypes differed by one substitution from the central haplotype (H1), and one haplotype (H7) differed by two substitution steps (Figure 4, Table 3). Notably, the northern sites (NW1 and NW2) were monomorphic and fixed for the dominant H1 haplotype.

## 4. Discussion

The occurrence of *B. bufo* in highly urbanized environments has been well-documented, including previous surveys in Warsaw (e.g., [30,42,44,58,60]). This study examined the genetic structure of common toads in a strongly fragmented urban landscape and revealed a pattern indicating a temporal mismatch between ecological isolation and genetic differentiation. Specifically, all sites exhibited heterozygosity deficits and positive F_IS_ values, whereas pairwise F_ST_ values remained generally low despite being frequently significant. This combination of within-population genetic erosion and only weak among-population divergence is consistent with a post-fragmentation genetic inertia (time-lag effect).

Genetic diversity across the sampling sites was relatively low, accompanied by a heterozygosity deficit and consistently positive inbreeding coefficients (F_IS_ up to 0.246), likely reflecting disruption of mating structure and within-site substructuring reinforced by strong breeding-site fidelity [31,32]. These patterns do not exclude the possibility of reduced gene flow among populations, consistent with known effects of habitat fragmentation in urban landscapes [29,30]. In contrast, pairwise F_ST_ values (Figure 2) remained low, albeit significant, suggesting that spatial genetic structure is still weakly developed.

Taken together, these results are consistent with a post-fragmentation genetic inertia (time-lag effect; [7,28]), reflecting a temporal decoupling between within-population processes (e.g., inbreeding, heterozygosity decline) and among-population divergence (F_ST_). Such a lag phase occurs when the time since fragmentation is too short for genetic drift to have produced strong differentiation, especially in species with relatively long generation times or delayed generation turnover [7], as is the case of this species due to delayed sexual maturity [84,86]. No bottleneck signals were detected, suggesting that relatively large effective population sizes may currently buffer the genetic consequences of isolation and delay detectable genetic erosion.

However, this genetic lag is a critical concern for conservation; while populations appear only weakly differentiated today, the reduced allelic richness and effective population size (Ne) observed in the northern sites (NW1/NW2) may represent the leading edge of genetic erosion. This suggests that landscape isolation is already translating into demographic impacts that precede full genetic divergence. As gene flow remains limited, drift and inbreeding are expected to intensify over time, particularly in ponds embedded within highly resistant urban infrastructure. It should be noted that the northern sites (NW1 and NW2) were represented by relatively small sample sizes, reflecting the low abundance of toads in the field. Consequently, genetic parameter estimates for these sites are associated with greater uncertainty and should be interpreted with appropriate caution.

Ecologically, the six ponds analyzed are clearly isolated, surrounded by roads, buildings, and artificial barriers lacking safe dispersal corridors. Movement is further limited by the low vagility of *B. bufo* (~200–300 m mean dispersal), road mortality, and strong breeding-site fidelity. These ecological constraints are therefore already in effect, even though their genetic consequences remain delayed—a pattern widely reported in landscape genetics and metapopulation theory [7,28]. The interpretation of our results as a genetic time-lag effect is also consistent with the expected temporal scale of urban fragmentation in Warsaw. Although the exact onset of isolation differs among sites, major urban expansion and road-network development affecting many of the studied districts occurred primarily during the second half of the twentieth century. Given the estimated generation time of *B. bufo* (approximately 5–7 years; [84,86]), the populations examined here have likely experienced only several to a dozen generations under altered connectivity regimes. Simulation studies indicate that this is precisely the period during which genetic consequences of fragmentation begin to emerge, while strong differentiation may still remain weak or incomplete [28]. The combination of heterozygosity deficits, positive F_IS_ values, significant but low F_ST_, and the absence of bottleneck signatures observed in our study is therefore consistent with populations currently occupying an intermediate stage between ecological isolation and fully developed genetic divergence.

Although a legacy of historically higher connectivity may still obscure ongoing isolation, potentially facilitated by formerly more continuous riparian and semi-natural habitats within the Vistula valley, continued restriction of dispersal is expected to progressively alter genetic structure. Furthermore, the present study cannot fully exclude the possibility that part of the observed genetic structure reflects historical population subdivision predating recent urban development. Distinguishing between pre-existing genetic gradients and fragmentation-induced divergence would require temporal samples or comparable data from populations inhabiting the region prior to large-scale urban expansion, which are currently unavailable for the Warsaw system.

While we interpret our results as consistent with post-fragmentation genetic inertia, alternative hypotheses should be considered. The lack of strong genetic structure could reflect cryptic gene flow via green corridors, or selective filtering imposed by urban environments may also be contributing to the homogenization of genotypes through differential survival. Additionally, high intra-pond reproductive variance may obscure existing structure. Disentangling these mechanisms will require integrative approaches combining spatial ecology, demographic data, and genomic analyses.

Urban infrastructure, particularly roads, likely restricts migration from non-urban areas, while fragmented landscapes constrain dispersal to breeding sites [87]. Notably, we found no significant F_ST_ division across the Vistula River, suggesting that this large natural feature does not constitute a major phylogeographic barrier for *B. bufo* in Warsaw, in contrast to patterns reported in other systems [53,54]. Instead, anthropogenic barriers appear to play a dominant role. For example, despite their proximity, the ponds at sites NW1 and NW2 are effectively isolated by roads. Such separation likely exacerbates dispersal constraints, particularly when breeding and hibernation sites are divided [88].

Despite the low pairwise genetic distances (F_ST_) between sites, their statistical significance suggests non-random differentiation. The greatest distances were observed between the two localities with the highest number of haplotypes (SE2 and E) and those with the lowest (NW1 and NW2). Clustering analyses using STRUCTURE and BAPS further supported this pattern, grouping all multi-haplotype localities (SE1, SE2, W, and E) separately from single-haplotype sites. The differences between STRUCTURE, BAPS and DAPC likely reflect the distinct assumptions and sensitivities of these methods. STRUCTURE is known to have limited power to detect weak genetic structure when differentiation is low and the number of loci is small, whereas DAPC is specifically designed to maximize among-group variation and is often more sensitive under such conditions. BAPS, in turn, tends to identify broader genetic partitions. Therefore, we interpret the concordant separation of northern sites detected by both BAPS and DAPC as evidence of biologically meaningful differentiation, while the weaker STRUCTURE signal is consistent with the overall low levels of genetic divergence observed in the dataset.

Overall, the clustering analyses do not support the existence of strongly differentiated genetic units within Warsaw. Instead, the convergent signal across STRUCTURE, BAPS, snapclust, and DAPC indicates weak and spatially heterogeneous differentiation, consistent with an urban population currently undergoing fragmentation rather than a system composed of well-defined genetic clusters.

Across geographically distant populations (Norway, Britain, and Warsaw), a broadly similar pattern emerges: a single dominant cytochrome *b* haplotype accompanied by multiple rare variants, typically restricted to one or a few individuals. In our study, one haplotype accounted for approximately 80% of individuals, a pattern comparable to that reported in Norway [89] and Britain [90], despite substantial differences in geographic scale and sample size. In the British dataset, the dominant haplotype (B1) differed from our H1 by only a single substitution and has also been reported from continental Europe (France, Belgium, Germany, Hungary), indicating extremely shallow divergence and a shared postglacial history.

However, while mitochondrial data reflect this common evolutionary background, nuclear microsatellite markers reveal divergence in contemporary population processes. In contrast to British populations, where genetic structure is primarily shaped by isolation-by-distance and reflects a relatively stable equilibrium, the Warsaw population exhibits features of a system in transition. Specifically, we observed consistently positive inbreeding coefficients (F_IS_ up to 0.246), a heterozygosity deficit across most sites, and low but significant F_ST_ values among sampling locations, alongside relatively high or unbounded Ne and no evidence of recent bottlenecks. This suggests that demographic buffering may temporarily maintain genetic diversity despite reduced connectivity. As a consequence, the emergence of strong spatial genetic structure may be delayed.

Together, these results suggest that the Warsaw population may represent a transient stage following recent habitat fragmentation, in which historical genetic variation is still retained despite emerging signals of inbreeding and altered mating structure. In this phase, populations remain genetically similar at the regional scale, while local processes begin to reshape genetic variation.

The mitochondrial and nuclear datasets capture processes operating at different temporal scales. The star-like mtDNA haplotype network, low nucleotide diversity, and shallow divergence among haplotypes are consistent with a common postglacial origin and subsequent expansion of the studied populations. In contrast, microsatellite markers revealed weak but detectable population differentiation, particularly among the northern sites. This discrepancy is consistent with the different temporal resolutions of the two marker systems, with mtDNA reflecting historical demographic processes and microsatellites being more sensitive to recent restrictions in gene flow. Together, these results suggest that contemporary urban fragmentation is superimposed on a largely shared historical genetic background. The persistence of low mitochondrial differentiation alongside emerging nuclear structure is also consistent with a time-lag between ecological isolation and the accumulation of stronger genetic signatures of fragmentation.

In a complementary analysis, we applied supervised feature selection (MCFS-ID) to identify genetic and phenotypic variables distinguishing individuals across sites. This analysis was not intended to assess genetic isolation directly, but rather to determine whether site differentiation was accompanied by consistent ecological or morphological differences among individuals. Although this approach does not validate population structure, it highlights features potentially associated with ecological divergence. Among phenotypic traits, body mass varied significantly between sites, possibly reflecting differences in resource availability or demographic structure [91]. Environmental disturbances [35] affecting either larval recruitment or adult survival may further shift the overall size structure of these urban populations, explaining the variation in body mass distribution [42]. The reduced body mass observed in northern populations may also reflect differences in habitat quality, resource availability, or exposure to urban stressors such as pollution. Although the present data do not allow inference of adaptive responses, ecological effects of urbanization may emerge before strong genome-wide genetic differentiation becomes detectable, particularly under a scenario of genetic inertia. Genetically, several microsatellite alleles—most notably allele 186 at locus Bbuf49—showed high discriminatory power. While microsatellites are generally considered neutral, such patterns may reflect allele-frequency differences generated by drift, demographic history, or linkage to genomic regions affected by selection; distinguishing among these mechanisms would require further investigation using genome-wide approaches (e.g., RAD-seq). Notably, based on MCFS-ID results, genetic features contributed little to discrimination between sexes, whereas morphological and reproductive traits accounted for most of the observed differences.

Overall, our findings support the presence of a delayed genetic response to recent fragmentation, in which subtle genetic differentiation masks ongoing ecological isolation imposed by a fragmented urban matrix. *B. bufo* populations in Warsaw currently retain substantial genetic variation despite increasing fragmentation; however, without restoration of functional connectivity, continued restriction of gene flow is likely to lead to increased inbreeding, genetic drift, and reduced adaptive potential. The integration of landscape genetics insights into urban planning is crucial: prioritizing the establishment of functional green corridors and low-resistance pathways (such as underpass systems) is essential to lower the effective resistance of the urban matrix. In Warsaw, efforts aimed at maintaining or restoring connectivity among currently isolated breeding sites, including habitat continuity within the Vistula River corridor, may help counteract the long-term genetic consequences of fragmentation. In a rapidly developing capital like Warsaw, such actions are vital to ensure the long-term resilience and connectivity of amphibian biodiversity in urban environments. Repeated genetic surveys conducted over the coming decades would also provide a valuable opportunity to assess whether the currently inferred phase of genetic inertia is followed by increased population differentiation and further loss of genetic diversity.

## 5. Conclusions

Our findings indicate that urban populations may retain substantial genetic variation despite habitat fragmentation, at least during the early stages of genetic response to landscape change. In Warsaw, *B. bufo* appears to maintain considerable genetic diversity despite substantial landscape fragmentation, consistent with a time-lag between ecological isolation and detectable genetic erosion. However, this stability is likely transient; without timely conservation interventions, the population may move beyond the current lag phase toward progressive drift-driven loss of variation. Consistent with broader findings from landscape genetics and amphibian conservation studies, maintaining functional connectivity through green corridors and low-resistance dispersal pathways (e.g., underpasses) may help sustain population resilience and long-term evolutionary potential in urban environments.

## Figures and Tables

**Table 1:** Genetic variability of *B. bufo* populations across sampling sites.

Sampling Sites									
Variables	W	E	SE1	SE2	NW1	NW2	Statistic	*p*-Value	Test
Sample size	18	21	20	19	9	10			
Na	6.143	6.429	6.714	7.000	4.286	4.000	4.213	0.519	KW
Ar	4.713	4.813	4.924	5.230	4.046	3.720	1.571	0.905	KW
Ho	0.488	0.552	0.504	0.627	0.437	0.400	5.093	0.405	KW
He	0.574	0.649	0.612	0.614	0.474	0.497	0.570	0.724	A
F_IS_	0.179	0.174	0.202	0.005	0.138	0.246	0.870	0.514	A
HWE	**HD**	**HD**	**HD**	HD	**HD**	**HD**			
*p*-value	**0.0333**	**0.009**	**0.006**	0.160	**0.042**	**0.041**			
No. Private Alleles	0.714	0.571	0.429	1.000	0.429	0.143			
Ne LDNe									
Pcrit = 0.02	−100.90	77.90	834.20	−2387.80	−26.30	36.50			
CI	47.5–inf	24.3–inf	38.9–inf	39.6–inf	12.5–inf	4.2–inf			
Pcrit = 0.05	−61.10	38.70	−1606.60	−165.10	−26.30	36.50			
CI	47.2–inf	13.2–inf	34.1–inf	40.8–inf	12.5–inf	4.2–inf			
Ne NeEstimator									
Pcrit = 0.02	infinite	67.60	738.80	481.10	infinite	36.50			
CI	46–inf	23.0–inf	38.6–inf	35.7–inf	12.0–inf	4.2–inf			
Pcrit = 0.05	infinite	34.90	infinite	infinite	infinite	36.50			
CI	45.6–inf	12.6–inf	33.9–inf	36.7–inf	12.0–inf	4.2–inf			
Bottleneck TPM 90%	0.813	0.406	0.813	0.500	0.980	0.852			
SMM	0.961	0.813	0.945	0.945	0.988	0.973			

Na—mean number of alleles, Ar—allelic richness, Ho—observed heterozygosity, He—expected heterozygosity, F_IS_—inbreeding coefficient, HWE—Hardy–Weinberg equilibrium test (HD—heterozygote deficit); No. Private Alleles—mean number of private alleles; Ne—effective population size calculated using a linkage disequilibrium method; LDNe—LDNe software v1.31, NeEstimator—NeEstimator software v2.1; TPM 90%—two-phase model with 90% of SMM; SMM—stepwise mutation model; KW—Kruskal–Wallis test; A—ANOVA. Statistically significant results were bolded. Confidence intervals for Ar, Ho, He, and F_IS_ are provided in Table S4.

**Table 2:** Genetic variability among designated clusters: putative landscape-induced subpopulations.

		Clusters					
Variables		1	2	3	Statistic	*p*-Value	Test
N		30	32	35	0.170	*p* > 0.10	Chi
Na		8.286	7.571	8.857	0.373	0.830	KW
Ar		8.139	7.205	8.249	0.423	0.809	KW
Ho		0.570	0.440	0.520	0.420	0.665	A
He		0.640	0.510	0.580	0.310	0.735	A
F_IS_		0.111	0.134	0.101	0.030	0.975	A
HWE		**HD**	HD	**HD**			
*p*-value		**0.048**	0.775	**0.010**			
No. Private Alleles		0.857	0.857	1.429			
Ne LDNe							
Pcrit = 0.02	0.02	233.400	134.400	116.600	19.450		
Pcrit = 0.05	0.05	233.800	30.000	47.700	55.640		
Ne NeEstimator							
Pcrit = 0.02	0.02	204.400	123.200	113.300	13.480		
Pcrit = 0.05	0.05	203.300	28.300	46.600	79.170		
Bottleneck TPM 90%		0.813	0.988	0.988			
SMM		0.973	0.988	0.996			

N—number of samples, Na—mean number of alleles, Ar—allelic richness, Ho—observed heterozygosity, He—expected heterozygosity, F_IS_—inbreeding coefficient, HWE—test for Hardy–Weinberg equilibrium (HD—heterozygote deficit); No. Private Alleles—mean number of private alleles; Ne—effective population size calculated using a linkage disequilibrium method; LDNe—LDNe software v1.31, NeEstimator—NeEstimator software v2.1; TPM 90%—two-phase model with 90% of SMM; SMM—stepwise mutation model; Chi—chi-squared test; KW—Kruskal–Wallis test; A—ANOVA. Statistically significant results were bolded.

**Table 3:** Number of haplotypes and their diversity across the sampling sites.

Population	Number of Individuals	Number of Haplotypes (h)	Haplotype Diversity (Hd)	Average Number of Differences (K)	Nucleotide Diversity (Pi)
W	18	2	0.294	0.294	0.00037
E	21	4	0.481	0.533	0.00067
SE1	20	3	0.279	0.368	0.00046
SE2	19	5	0.579	0.760	0.00095
NW1	9	1	0	0	0
NW2	10	1	0	0	0

## Data Availability

The mitochondrial DNA sequences generated in this study have been deposited in GenBank under accession numbers PQ463790–PQ463796. Raw microsatellite genotype data used for all population genetic analyses are provided in the Supplementary Materials (Table S7). Detailed information regarding microsatellite loci, allele scoring, population genetic analyses, clustering procedures (STRUCTURE, BAPS, DAPC, and snapclust), effective population size estimation, bottleneck analyses, and MCFS-ID feature selection analyses is provided either in the main text or in the Supplementary Materials. All data required to reproduce the results presented in this study are available within the article and its Supplementary Materials.

## Supplementary Materials

The following supporting information can be downloaded at: https://www.mdpi.com/article/10.3390/ani16131983/s1, File S1: Interactive map of common toad sampling sites in Warsaw (colored dots); Figure S1: BIC values and the number of clusters generated by the find.cluster function in the adegenet package in R. Three clusters were chosen as the optimal number and used for the analysis; Figure S2: MCFS-ID analysis results showing the relative importance (RI) ranking of attributes that distinguished groups between (A) sampling sites, (B) clusters, and (C) sex. The significant features are marked in green; Figure S3: Alleles that contributed the most to the obtained population structure. The loadings of the original alleles (variables) to the principal components of the DAPC; Figure S4: Cross-validation results for different classification models depending on the number of top selected attributes using MCFS-ID: (A) sampling sites (five significant features); (B) clusters (seven significant features); (C) sex (one significant feature). The X-axis represents the number of selected features, while the Y-axis shows the weighted accuracy (wacc) in percentage. Colors indicate different models. j48—Decision Tree (C4.5 implementation); random forest—Ensemble of Decision Trees; Naïve Bayes—Probabilistic Classifier based on Bayes’ Theorem; SVM—Support Vector Machine; kNN—k-Nearest Neighbors; logistic—Logistic Regression; ripper—Repeated Incremental Pruning to Produce Error Reduction; Table S1: Genetic and phenotype features of common toads; Table S2: Pairwise FST values among sampling sites with exact *p*-values and multiple-testing correction. Classification results based on the features found significant by MCFS-ID; Table S3: Hierarchical AMOVA testing the effect of Vistula River bank on genetic structure; Table S4: Confidence intervals for genetic diversity estimates of *Bufo bufo* populations across sampling sites; Table S5: Classification results based on the features found significant by MCFS-ID; Table S6: Haplotype-defining variants across mitochondrial positions; Table S7: Microsatellite genotypes of sampled individuals.
