# Supplementary figures and images for: Genetic Inertia in Urban Populations of the Common Toad (Bufo bufo): Evidence from Nuclear and Mitochondrial DNA
Source: Animals (Basel). 2026 Jun 27;16(13):1983. doi: 10.3390/ani16131983 (PMC13359437; doi:10.3390/ani16131983)

**Value of BIC  
versus number of clusters**

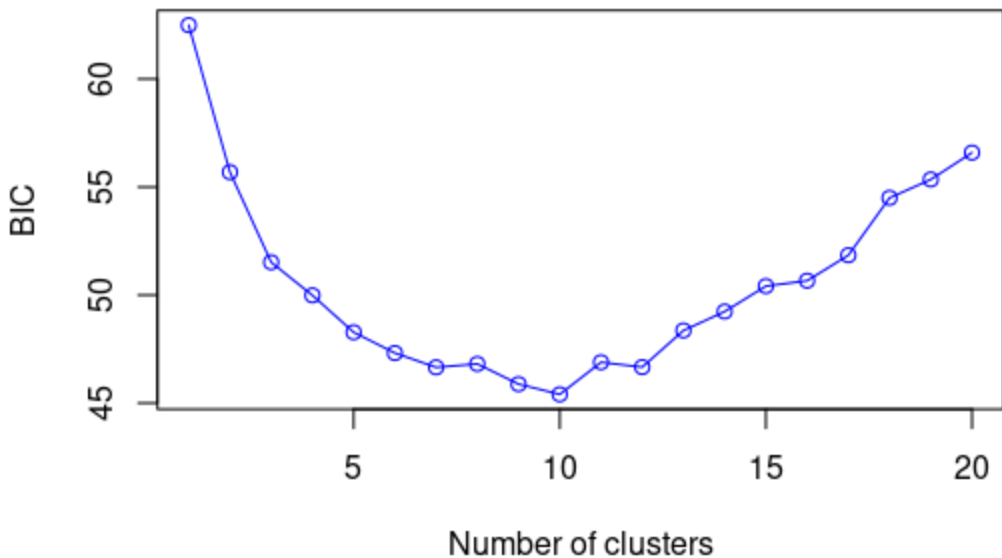

Supplement: Supplementary file 1 [file animals-16-01983-s001.zip › Figure S1.pdf]

# A

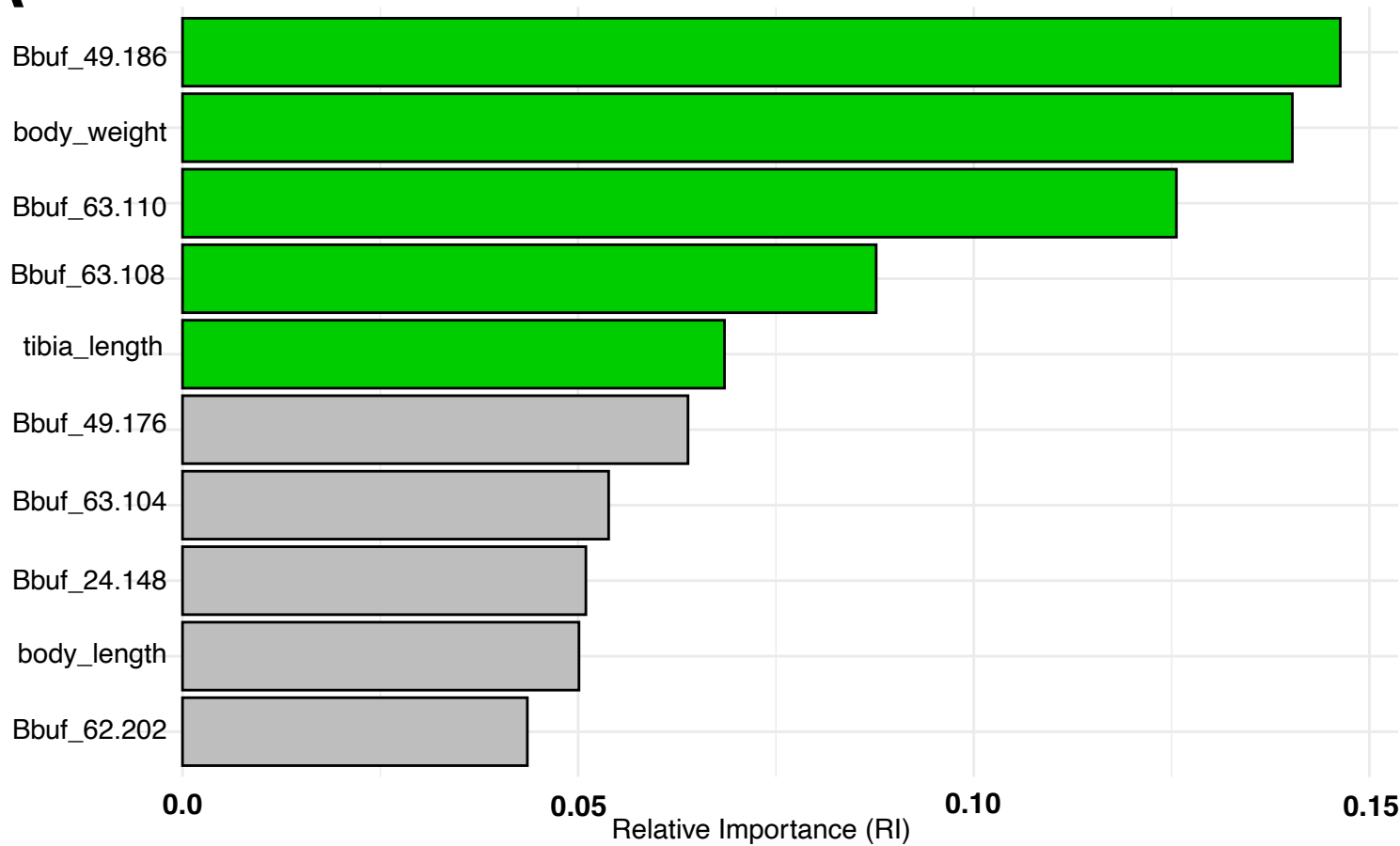

**B**

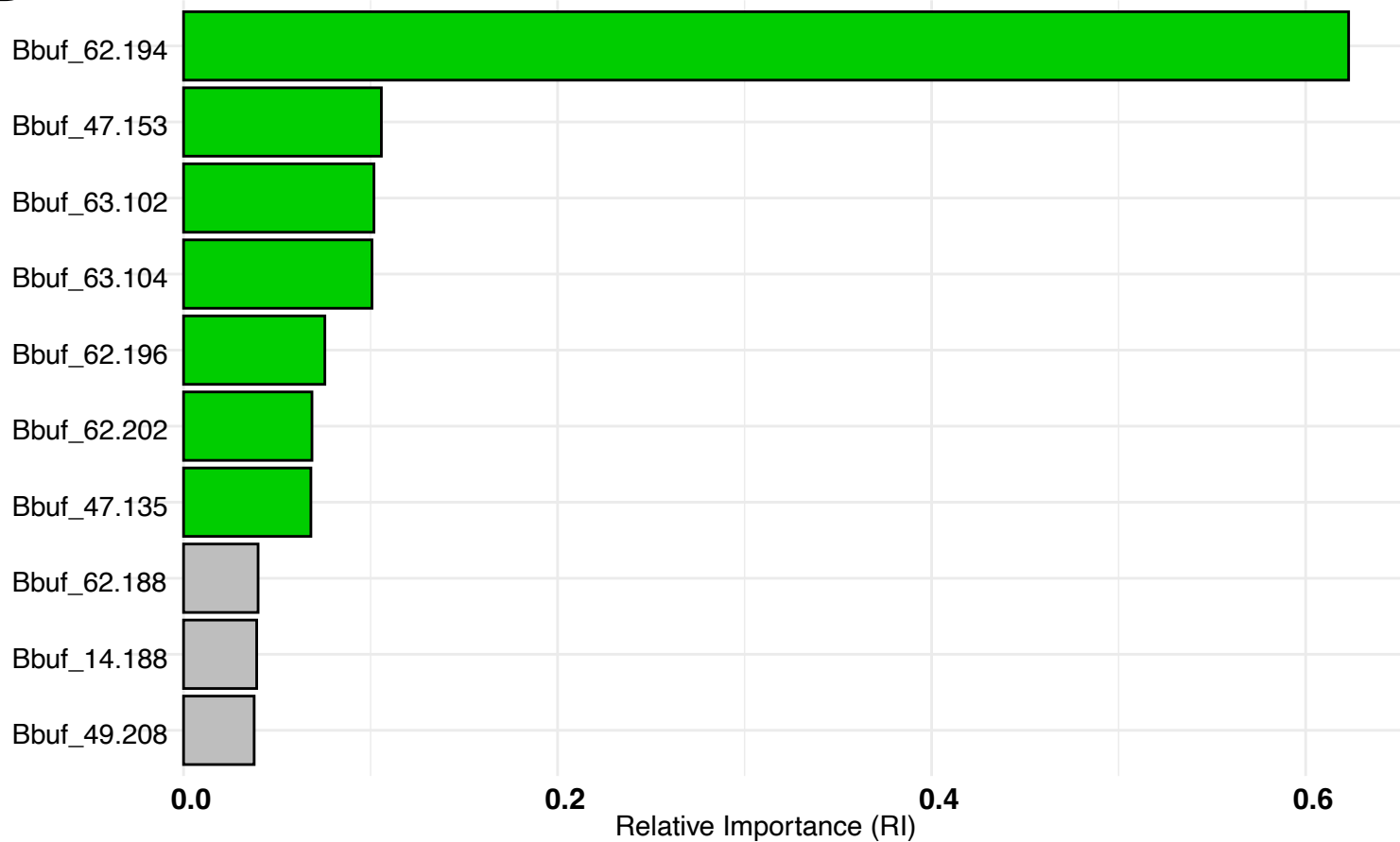

C

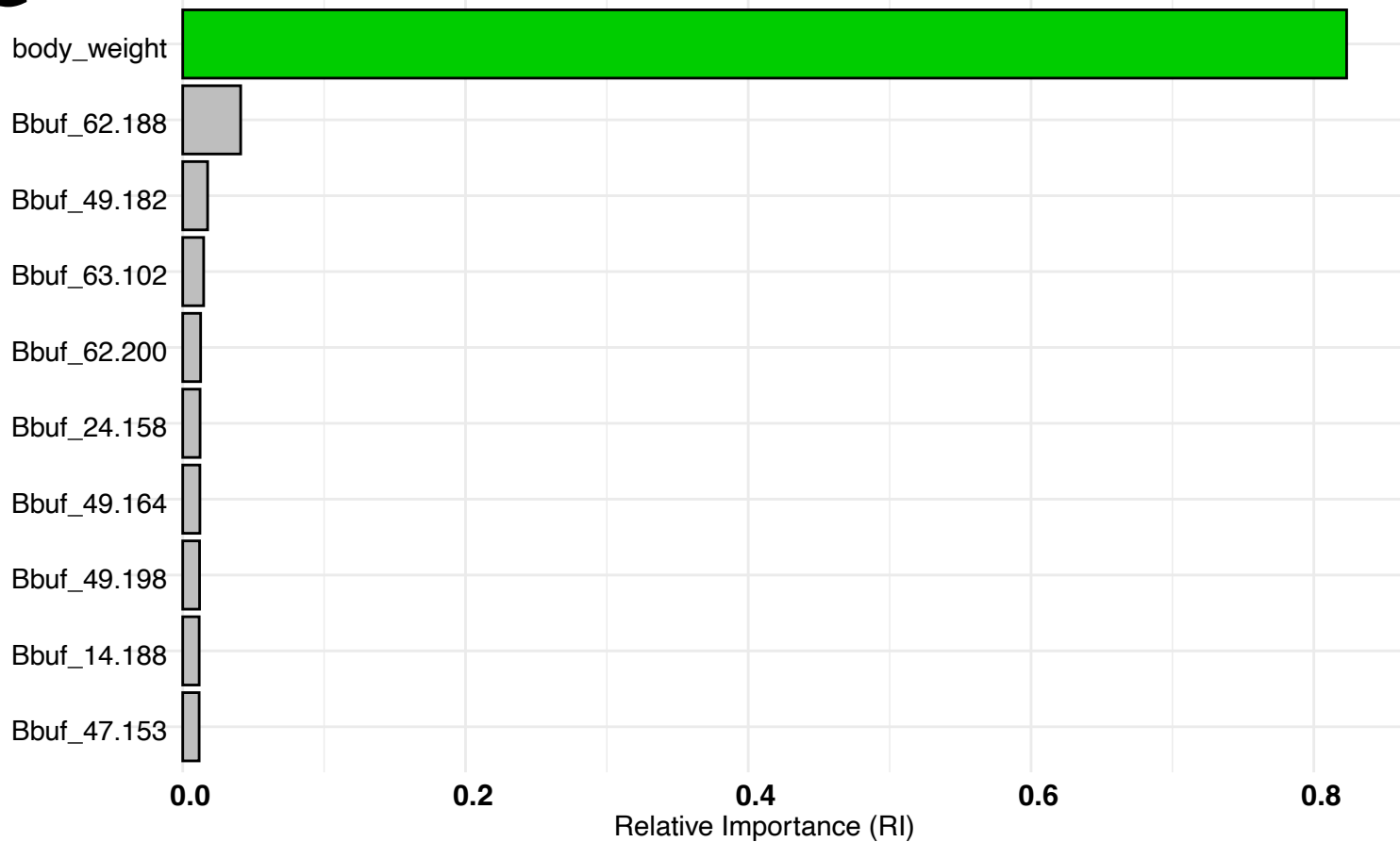

Supplement: Supplementary file 1 [file animals-16-01983-s001.zip › Figure S2.pdf]

# ALLELE FREQUENCY

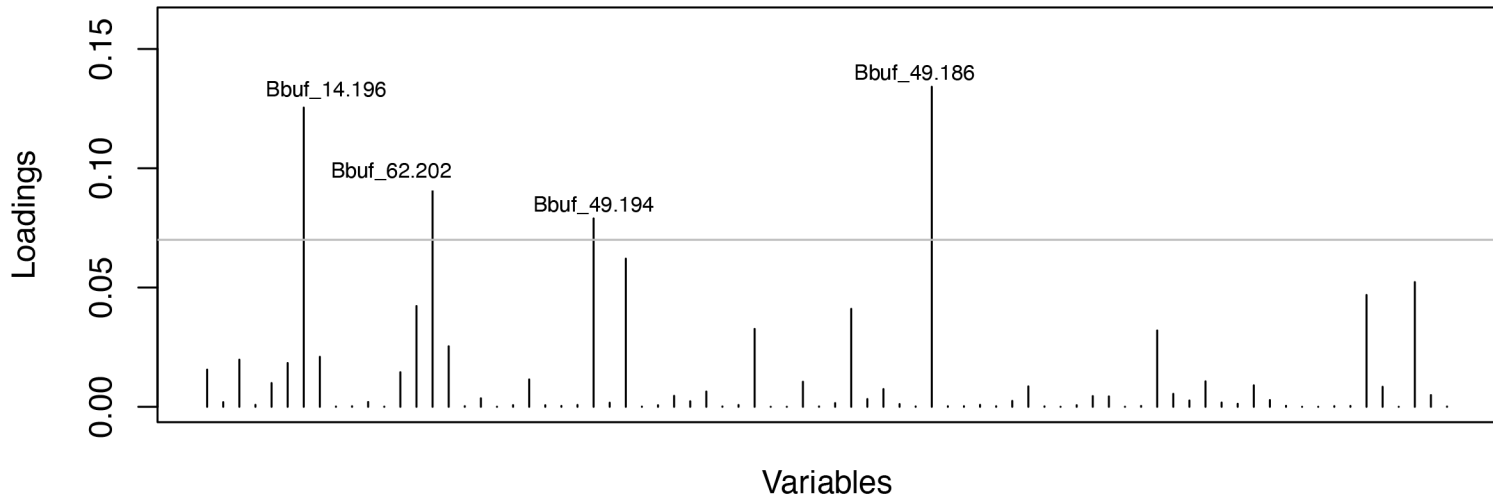

Supplement: Supplementary file 1 [file animals-16-01983-s001.zip › Figure S3.pdf]

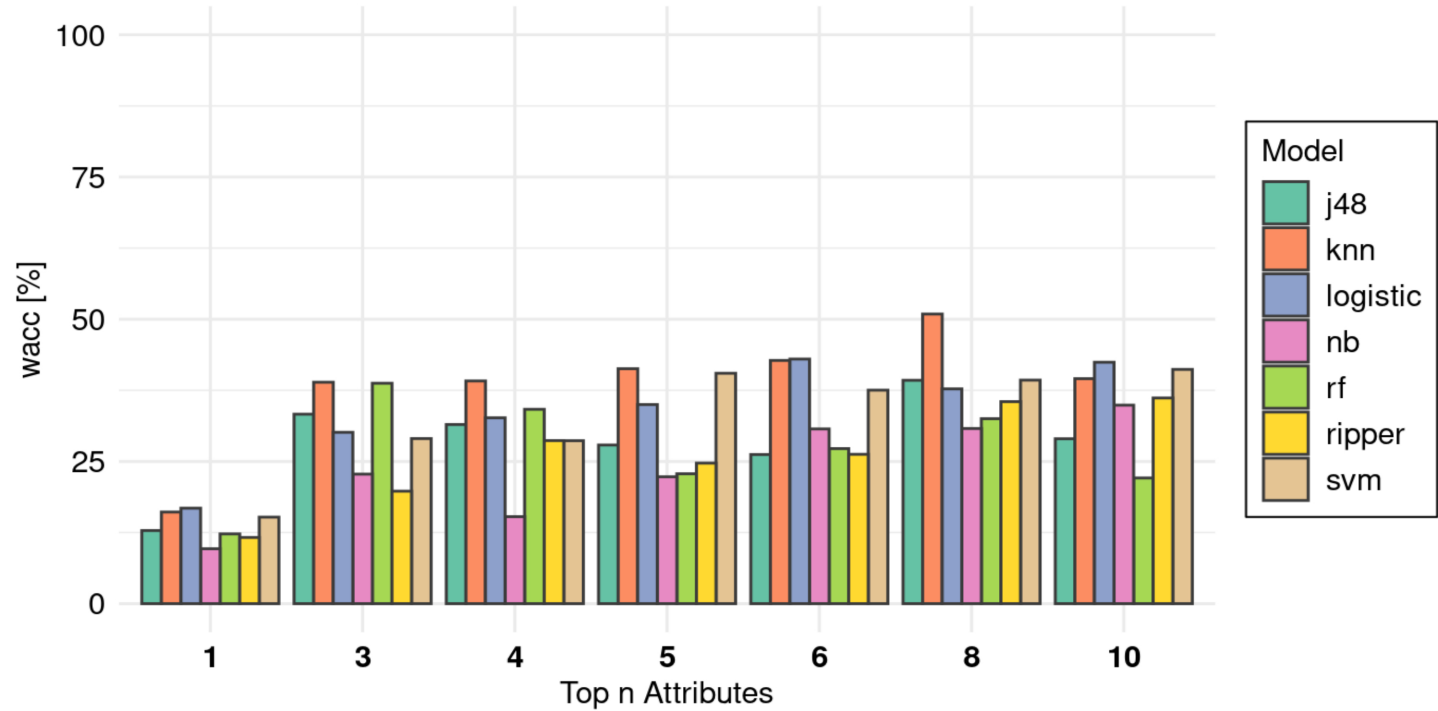

**Cross Validation Results (wacc)**

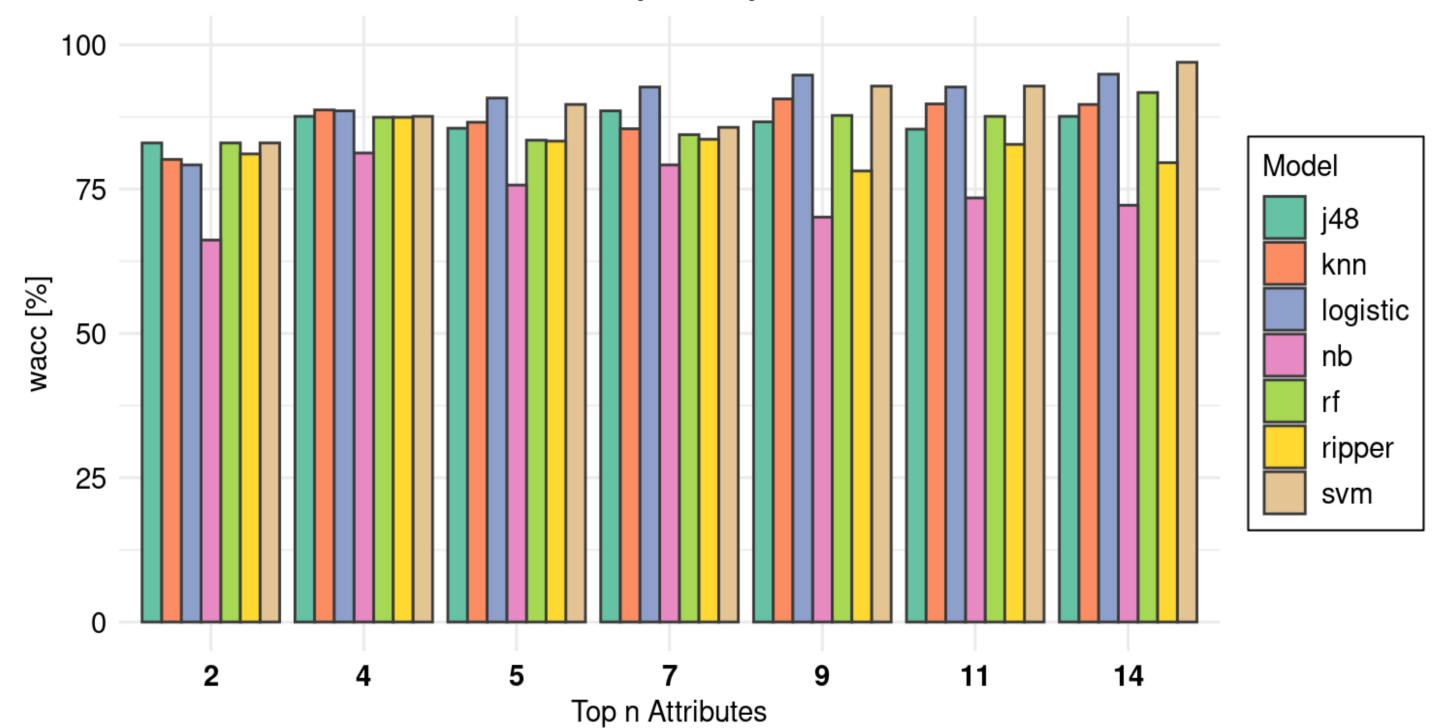

**Cross Validation Results (wacc)**

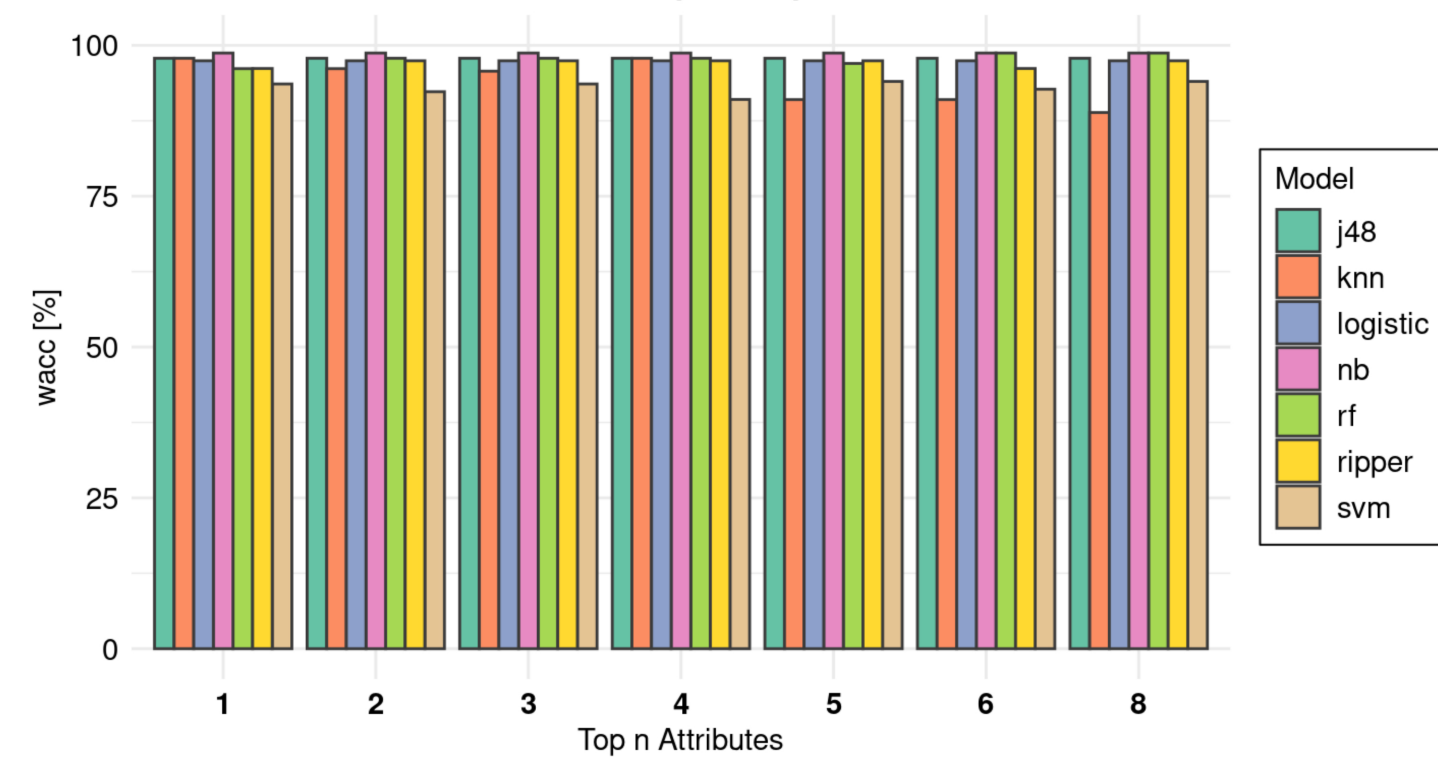

Supplement: Supplementary file 1 [file animals-16-01983-s001.zip › Figure S4.pdf]
